# Mobitz type II second-degree atrioventricular block: a commonly overdiagnosed and misinterpreted arrhythmia

**DOI:** 10.3389/fcvm.2024.1450705

**Published:** 2024-08-29

**Authors:** S. Serge Barold, Bengt Herweg

**Affiliations:** ^1^ Department of Medicine, University of Rochester University School of Medicine and Dentistry, Rochester, NY, United States; ^2^ Department of Medicine, Morsani College of Medicine, University of South Florida, Tampa, FL, United States

**Keywords:** Mobitz type II atrioventricular block, second-degree atrioventricular block, vagal tone, vagally induced atrioventricular block, Wenckebach atrioventricular block, cardiac pacemaker

## Abstract

Mobitz type II second-degree atrioventricular block (AVB) is an electrocardiographic pattern that describes what appears to be an all-or-none conduction without visible changes in the AV conduction time or PR intervals before and after a single non-conducted *P* wave. An unchanged PR interval after the block is a sine qua non of Mobitz type II block. A 2:1 AVB cannot be classified in terms of type I or type II AVB. The diagnosis of Mobitz type II block AVB requires a stable sinus rate, which is an important criterion because a vagal surge (generally benign) can cause simultaneous sinus slowing and AV nodal block, which can resemble Mobitz type II AVB. Atypical forms of Wenckebach AVB may be misinterpreted as Mobitz type II AVB when a series of PR intervals are constant before the block. Concealed His bundle or ventricular extrasystoles may mimic both Wenckebach and/or type II AVB (pseudo-AVB). Correctly identified Mobitz type II AVB is invariably at the level of the His–Purkinje system and is an indication for a pacemaker.

## Historical aspects

In 1906, John Hay from Liverpool, England, using recordings of the a–c interval from the venous pulse, described a new form of second-degree atrioventricular block (AVB) now considered to be Mobitz type II second-degree AVB (1). Using the jugular venous pulse, Hay observed no progressive changes of the a–c interval (corresponding to atrial and ventricular contractions, respectively) prior to an “a” wave that was not followed by a “c” wave.

**Figure 1:**
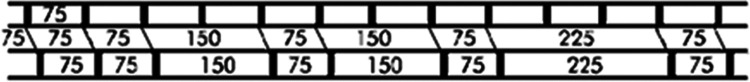
Diagram from Mobitz's 1924 article. Note that all the PR and PP intervals are constant. This pattern corresponds to the presently accepted definition of Mobitz type II atrioventricular block, except that it now considers the block of only one *P* wave.

In 1924, Mobitz, using the electrocardiograph, classified the well-known Wenckebach form of second-degree AVB as type I and characterized the form of AV block described by Hay as type II second-degree AVB in terms of “the occasional block of one or more *P* waves with no change in the PR interval before and after the non-conducted *P* waves” (2, 3). This definition with a relatively minor modification remains clinically useful to this day (4). The diagrams in Mobitz's original paper clearly show constancy of the sinus rate before and after the block, which is an important feature still commonly ignored (Figure 1). Mobitz commented that type II AVB often progressed to complete AVB and was associated with Stokes–Adams attacks and death (2). Mobitz did not indicate that sustained 2:1 AV block was a form of type II AVB; rather, he showed that a 2:1 AVB may be associated with sequences showing intermittent typical type II AVB. Unfortunately to this day, sustained 2:1 AVB is not infrequently called Mobitz type II AVB in the literature.

Mobitz believed that conduction in type II AVB was an all-or-none phenomenon without visible changes in AV conduction time. Much later, it was demonstrated that Mobitz type II AVB is, in effect, Wenckebach AVB with increments in AV conduction so minuscule that they cannot be appreciated or measured by a standard electrograph but are demonstrable with very high-speed recordings (5). Although electrocardiographic recordings at 25 mm/s conceal the true physiology of Mobitz type II AVB, the resultant all-or-none ECG pattern remains immensely useful to localize the site of the block to the His–Purkinje system (6).

In 1955 Katz, Pick, and Langendorf from the famous Chicago School described Mobitz type II AVB as a condition that develops “without warning that is, it occurs after a series of beats with a constant PR prolongation” (7). Of note is that the required number of constant PR intervals before the blocked beat was not mentioned in this statement (7). The Chicago School's description of Mobitz type II AVB launched a new definition still found in many textbooks and articles as an “electrocardiographic pattern characterized by failure of a single impulse to conduct to the ventricles in the absence of antecedent lengthening of the PR interval (normal or prolonged).” This inappropriate definition of Mobitz type II AVB may describe a form of atypical type I AVB (8, 9). The Chicago School allowed the shortening of the post-block PR interval by 0.02 s for the diagnosis of Mobitz type II AVB, but this is no longer acceptable.

## Organizational definitions of type II second-degree AV block

**Figure 2:**
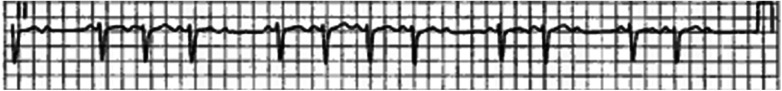
Mobitz type II AV block. There is a regular sinus rhythm with a single non-conducted *P* wave. The PR intervals before and after the block are constant. The RR interval encompassing the blocked *P* wave is twice the RR interval prior to the blocked *P* wave. From Barold SS. Definitions and pitfalls in the diagnosis of atrioventricular block. Heart Lung Circ. 2023;32:1413–1416.

According to the definitions codified by the World Health Organization (WHO) and the American College of Cardiology (both in 1978), Mobitz type II second-degree AVB is defined as the occurrence of a single non-conducted *P* wave associated with constant PR intervals before and after the blocked impulse, provided that the sinus rate or the *P*–*P* interval is constant (no slowing) and there are at least two consecutive conducted *P* waves (i.e., 3:2 AV block) to reveal the behavior of the PR interval (6, 10–12) (Figure 2) (Table 1). The current definition of Mobitz type II AVB is the same as Mobitz's original definition, except that it addresses only a single blocked *P* wave. An unchanged PR interval after the block is a sine qua non of type II block. The stability of the sinus rate with the absence of slowing is an important criterion because a vagal surge can cause simultaneous sinus slowing and AV nodal block (generally a benign situation) that can superficially resemble Mobitz type II AVB (13, 14). Mobitz type II AVB is not present if the *P*–*P* interval lengthens before or after the blocked beat (as in vagally induced block), even if the PR intervals remain constant before and after the block. In contrast, in Mobitz type II AVB, the pause encompassing the blocked *P* waves should equal two (*P*–*P*) cycles.

**Table 1:** Contemporary definition of type 2 second-degree AV block.

1. Single non-conducted P wave associated with constancy of all the PR intervals before and that of the conducted after the blocked impulse. 2. There are at least two consecutive conducted P waves (i.e., 3:2 AV block). 3. The sinus rate is constant. 4. Concealed extrasystoles must be excluded.

The above definition rules out a 2:1 or higher degree of second-degree AVB block, which can occur in either the AV node or the His–Purkinje system (15, 16). Unfortunately, a 2:1 AV block remains widely and erroneously called Mobitz type II AVB in the literature. Furthermore, during a stable sinus rhythm, if 1:1 AV conduction is followed by sudden AV block of several impulses (>1) with unchanged PR intervals, the purist will insist on calling this pattern as Mobitz type II AV block by citing the original description by Mobitz despite the accepted codified contemporary definition of Mobitz type II AVB that requires only one blocked beat for the diagnosis. Worse still, using the term “Mobitz block” presumably referring to Mobitz type II AVB is potentially misleading without specifying whether it is Mobitz type I or II AVB.

Mobitz type I and II AVBs are merely electrocardiographic patterns involving how single beats are blocked. Mobitz type II AVB may be transient, tachycardia-dependent, or permanent, but it is irreversible except in very rare circumstances (17). The commonest cause of Mobitz type II AV is age-related idiopathic fibrosis of the conduction system. Mobitz type II AVB is associated with bundle branch block in approximately 70–80% of cases. Mobitz type II AVB, as defined above, is universally accepted as being due to His–Purkinje disease and is an indication for a pacemaker, regardless of symptoms (18, 19).

Any form of narrow QRS AVB other than Wenckebach AVB should not be automatically called Mobitz type I AVB. Additionally, any form of wide QRS complex AVB other than Mobitz type II AVB should not be automatically labeled as Mobitz type II AVB. Infranodal block and infra-Hisian block are terms that refer to the anatomic location of the block, whereas Mobitz type II AVB refers to an electrocardiographic pattern associated with the block at these levels. The temptation of calling advanced block infranodal should also be avoided because the block may be in the AV node.

## Influence of heart rate on diagnosis

A modest increase or decrease in the sinus rate permits the diagnosis of Wenckebach AVB, but the sinus rate must be constant when PR intervals are measured to determine the presence of Mobitz type II AVB. However, Mobitz type II AVB, like any block in the His–Purkinje system, can be tachycardia-dependent so that its diagnosis can be made during an increasing sinus rate as with exercise. Increasing the heart rate by incremental atrial pacing may also precipitate Mobitz type II AVB in patients with His–Purkinje disease.

## Autonomic challenge and exercise

Carotid sinus massage and the administration of atropine have been advocated to distinguish Wenckebach AVB from Mobitz type II AVB. If the effect is greater on the sinus rate than the AV node, then atropine will increase the degree of block in the His–Purkinje system, and, conversely, vagal stimulation may reduce it. The use of autonomic maneuvers may be misleading, and they are seldom needed for diagnosis in clinical practice.

**Figure 3:**
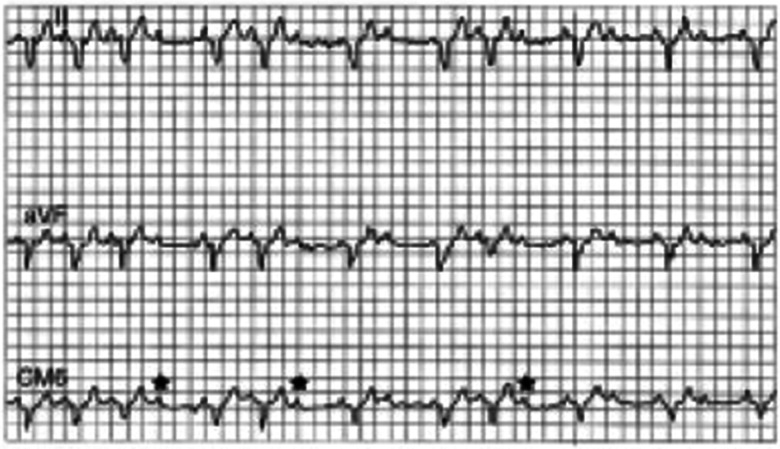
Type II atrioventricular (AV) block induced by exercise shown in a three-lead electrocardiogram (ECG) of a 68-year-old patient who had a near-syncope while playing tennis. Holter recordings were unremarkable. The ECG showed sinus rhythm at 60 beats/min, complete right bundle branch block, and left anterior hemiblock. During treadmill testing, the patient developed type II block (stars) at a rate of aproximately 95 per min, followed by a 2:1 AV block (right side of tracing). Related to SS, Hayes DL. Second-degree atrioventricular block: a reappraisal. Mayo Clin Proc. 2001 Jan;76(1):44-57. doi: 10.4065/76.1.44

Mobitz type II AVB commonly deteriorates to higher degrees of AVB on exercise, although, at times, exercise causes an increase in sympathetic tone so that Mobitz type II AVB may improve or disappear with exercise. Mobitz type II AVB may be precipitated by exercise, even when the basal ECG shows no evidence of AVB (Figure 3) (20–23).

## Vagally mediated AV block

**Figure 4:**
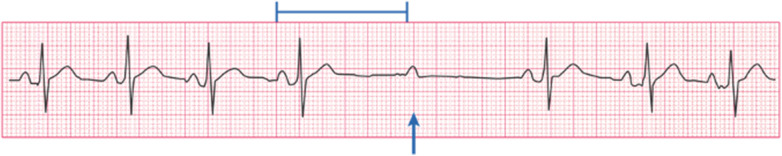
Vagally induced AV block. There is sinus slowing shown by the long PP interval. The PR intervals before and after a single blocked *P* wave are constantly simulating Mobitz type II block, which is, however, ruled out by sinus slowing. From Barold DC, Barold SS. ECG Simplified. Facts You Will Never Forget, Conductivity Press, San Marcos CA 2023.

Vagally mediated AVB is bradycardia-associated and not bradycardia-dependent. A vagal surge (generally a benign condition) causes simultaneous sinus bradycardia and depression of AV nodal conduction. In vagally induced Wenckebach AVB, the beat after the block may be followed either by a shorter or longer PR interval (escape beats) and sometimes by an unchanged PR interval (13, 14) (Figure 4). An unchanged PR interval after the blocked *P* wave may be due to a non-conducted *P* wave occurring fortuitously with an escape AV junctional beat so that the P–QRS relationship or PR interval is equal to that seen before the blocked *P* waves. Alternatively, a residual vagal effect on the AV node may prevent the expected PR shortening. The constancy of the PR intervals simulates Mobitz type II AVB. When this occurs, the clue to the diagnosis of vagally induced AV nodal block depends on the presence of sinus slowing. Sinus slowing may occur either before or after the blocked beat in association with a narrow QRS complex (13, 14).

Ignoring sinus slowing leads to the misdiagnosis of vagal Wenckebach AVB as Mobitz type II AVB. Accordingly, most of the erroneous case reports of the so-called Mobitz type II AVB are associated with sinus slowing. There is no mention of pacemaker implantation. Furthermore, most, if not all, of these cases are self-limited and appear benign in contrast to Mobitz type II AVB, which is irreversible and ultimately progresses to high-degree and complete AVB.

## Co-existence of type I and type II blocks

The presence of both narrow QRS Wenckebach AVB and Mobitz type II AVB-like patterns in a Holter recording, especially in patients with vagally induced AV block and sinus slowing, rules out the diagnosis of Mobitz type II AVB (24). The occurrence of both Wenckebach and Mobitz type II AVB in the same or separate ECGs is exceedingly rare and occurs only in patients with His–Purkinje disease (25, 26). However, rare forms of pseudo-Mobitz type II AVB may occur with concealed junctional and His–Purkinje extrasystoles. Therefore, when faced with the combination of Wenckebach AVB and Mobitz II AVB-like recordings, one can be almost certain that such a tracing is showing only Wenckebach AVB. Furthermore, follow-up monitoring will almost always reveal more obvious sequences characteristic of Wenckebach AVB.

## Drug-induced Mobitz type II AVB

1. Lithium. Armstrong et al. (27) reported two cases of lithium-associated Mobitz type II AVB. Both patients required placement of a permanent pacemaker due to persistent block and symptoms despite attempts at lowering the daily dose of lithium. The report presented no evidence that lithium was the cause of Mobitz type II AVB. In this respect, there are rare reports of first- and second-degree and complete AVB attributed to lithium sometimes with therapeutic doses (28–31). However, these anecdotal reports appear to be compromised by advanced patient age (predisposing to AVB), polypharmacy, and lack of appropriate follow-up, which is an important consideration because inherent AVB can be intermittent and may recur after a period of observation off medication (32). The site of AVB remains unknown.
2. Cocaine. There is a claim that cocaine can induce Mobitz type II AVB. However, the published ECG tracings clearly showed sinus slowing at the time of the block, therefore ruling out Mobitz type II AVB (33).
3. Antipsychotics. Naono et al. (17) reported a genuine case of convincing reversible Mobitz type II AVB in a 75-year-old patient with the right bundle branch block and first-degree AVB, who underwent treatment for schizophrenia with intramuscular haloperidol and levomepromazine. The medications were discontinued, and Mobitz type II AVB block disappeared after 3 days; however, the right bundle branch block during 1:1 conduction persisted.
4. Antiarrhythmic agents. In the past, an ajmaline challenge was used to evaluate the status of His–Purkinje disease. Recently, Roca-Luque et al. (34) performed electrophysiologic studies in patients with syncope, a wide QRS complex, and a His to ventricle (HV) interval <70 ms during incremental atrial pacing and the intravenous administration of flecainide or procainamide. Some patients developed second-degree infra-His or intra-His block. Although Mobitz type II AVB was not specifically stated, its occurrence must have been virtually certain.

## Potential errors in the diagnosis and treatment of second-degree type II AV block

**Figure 5:**
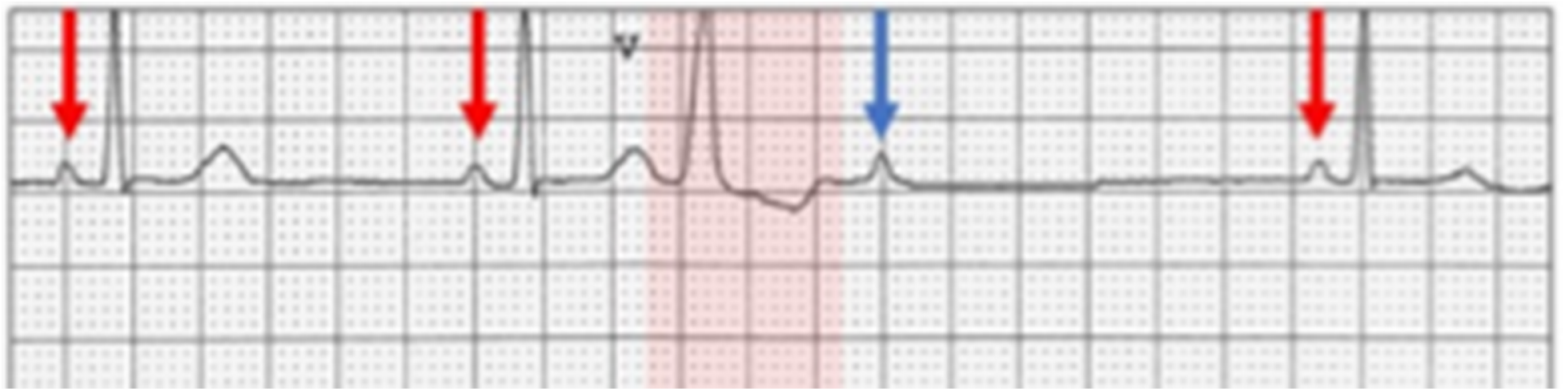
AVB caused by interpolated premature complex. Concealed retrograde conduction to the AV node from the VPC causes physiological AVB. The arrows point to the *P* waves. (Courtesy of Harry Mond, MD).

**Figure 6:**
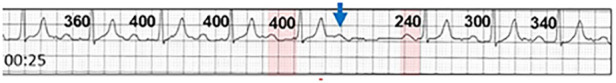
Atypical type I sequence with constant PR intervals (400 ms) for several beats before the block. The PR interval of the first conducted beat after the block is shorter. This pattern must not be interpreted as type II block. Intervals in milliseconds. From Barold SS. Definitions and pitfalls in the diagnosis of atrioventricular block. Heart Lung Circ. 2023;32:1413–1416.

**Figure 7:**
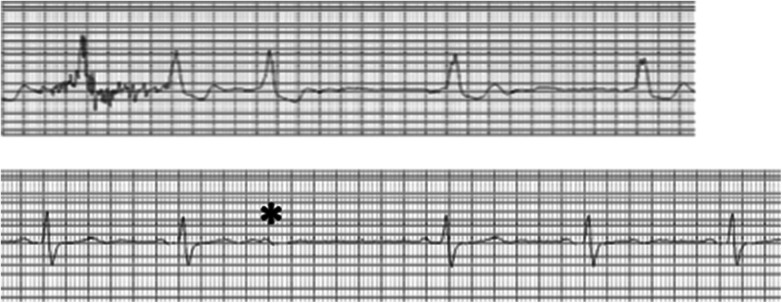
ECG strip from a monitor showing masking of Mobitz type II AVB by baseline interference. The top ECG strip shows how the diagnosis of Mobitz type II AVB can be suspected by observing multiple pauses (X2) of the sinus interval when *P* waves are not visible. When faced with such pauses, they should be considered the result of Mobitz type II AVB until proven otherwise. The bottom ECG strip shows that perseverance eventually provides visible *P* waves to make a diagnosis.

**Figure 8:**
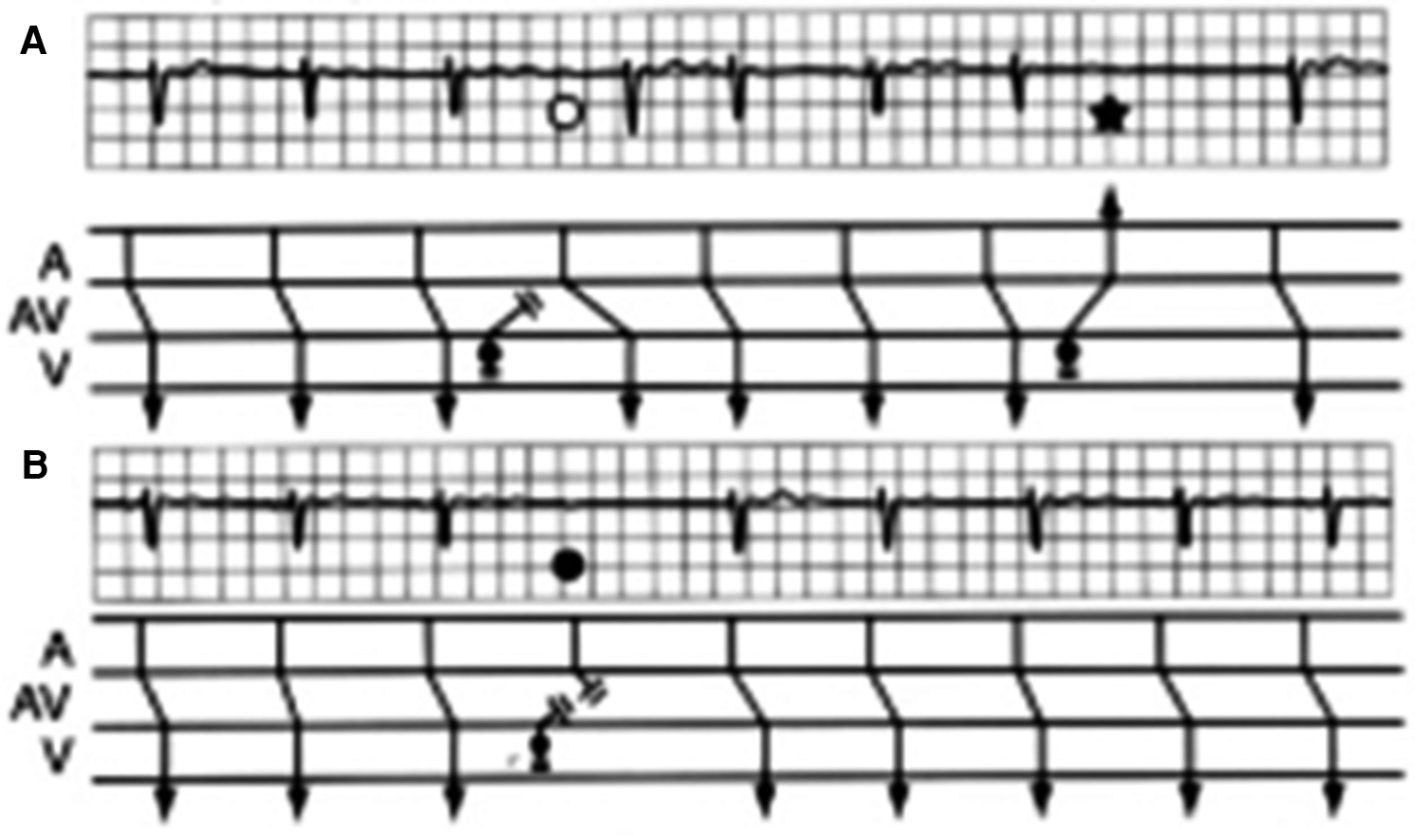
**(A)** Concealed extrasystole. There is a sinus rate at the time of block with constant PR intervals and importantly unchanged PR intervals before and after the blocked impulse, so that the pause is equal to two *P*–*P* intervals. The blocked *P* wave is sinus and not retrograde judged by its timing and configuration (solid circle). The concealed extrasystole penetrated the AV junction retrogradely. In this instance, the normal sinus impulse (solid circle) encountered a totally refractory AV node and was therefore blocked, a phenomenon simulating type II second-degree AV block. The tracings allow the confident diagnosis without invasive confirmation with His bundle recordings because true type II block can be ruled out by scrutiny of panel **B**. From Barold SS, Hayes DL. Second-degree atrioventricular block: a reappraisal. Mayo Clin Proc. 2001;76:44–57. **(B)** The fourth beat shows a sudden marked increase in the duration of the PR interval (open circle). A bidirectional block of the concealed extrasystole causes a sudden marked prolongation of the PR interval (without a change in the timing of sinus node discharge). The sinus impulse that follows the concealed extrasystole arrives in the AV node during its relative refractory period engendered by the previous retrograde conduction of the concealed extrasystole. Related to SS, Hayes DL. Second-degree atrioventricular block: a reappraisal. Mayo Clin Proc. 2001 Jan;76(1):44-57. doi: 10.4065/76.1.44

1. Non-conducted atrial premature beats masquerading as AVB.
2. Interpolated ventricular premature complexes (VPC) followed by AVB occur when there is concealed retrograde conduction from the VPC rendering the AV node refractory to the next atrial impulse. Ignoring the VPC, the pattern physiologically resembles that of Mobitz type II AVB because of the constant PR intervals before and after the blocked beat (Figure 5).
3. Making the diagnosis of Mobitz type II AVB without seeing the true AV conduction in the first beat after the block.
4. Failure to realize that type I and type II AVB almost never occur in the same ECG or Holter recording. Beware of type I sequence with tiny increments of AV conduction that mimic type II block, which appears to be extremely unlikely in association with obvious type I block.
5. Ignoring sinus slowing typical of vagally induced Wenckebach AVB. One should not diagnose Mobitz type II AVB, especially with a narrow QRS complex, if the *P*–*P* interval lengthens before and/or after the block, even if the PR intervals are constant before and after the block (13, 14, 35, 36).
6. Atypical Wenckebach AVB mistaken for Mobitz type II AVB. A current popular definition of Mobitz type II AVB found in many textbooks and articles describes it in terms of an “electrocardiographic pattern characterized by failure of a single impulse to conduct to the ventricles in the absence of antecedent lengthening of the PR interval whether it is normal or prolonged” (8, 9). Thus, Wenckebach AVB may be erroneously diagnosed as a Mobitz type II AVB when the terminal portion of a long sequence contains PR intervals with no discernible or measurable change before the blocked impulse (37). In such a case, the shortening of the PR interval of the first conducted beat after the block defines Wenckebach AVB (Figure 6).
7. Confounding ECG rhythm strips. When the *P* waves are not clearly visible, Mobitz type II AVB can be missed because the recording can be interpreted as showing sinus bradycardia or even sinus arrhythmia. With no visible *P* waves due to low amplitude or baseline noise artifact, the significance of pauses is mathematically equal to two sinus (PP) intervals, so typical of Mobitz type II AVB can be missed or misinterpreted as a 2:1 sinoatrial block (Figure 7). Such pauses should be considered representative of Mobitz type II AVB until proven otherwise, especially when associated with bundle branch block.
8. Medical emergency. The diagnosis of Mobitz type II AVB constitutes a medical emergency, even in minimally symptomatic patients with only short runs of AVB, because of the risk of sudden death by prolonged asystole or torsades de pointes (triggered by bradycardia) both ultimately capable of leading to ventricular fibrillation (38–41). Consequently, standby pacing should immediately be instituted either by temporary transcutaneous or transvenous pacing while waiting for the implantation of a pacemaker.
9. Concealed extrasystoles causing pseudo-AV block. Concealed extrasystoles may originate either from the AV junction or the His–Purkinje system and fail to propagate to the ventricular myocardium, although they may penetrate the AV junction (resetting AV junctional rhythms) and sometimes can conduct to the atrium (42–44). They are manifested by variable anterograde and retrograde conduction block. These concealed extrasystoles may produce a 2:1 AV block and a sudden appearance of a long PR interval due to concealed conduction and resemble conduction patterns of type I block and/or type II block (Figures 8A, B). There may be isolated retrograde *P* waves from retrograde conduction to the atrium simulating atrial premature beats. The diagnosis is suggested by the combination of apparent type I and type II sequences.

Many cases occur in patients with His–Purkinje disease. Interestingly, true type II block may actually co-exist with concealed extrasystoles in patients with His–Purkinje disease (20). Concealed extrasystoles may occasionally occur in patients without heart disease. Thus, in the presence of unexplained type II AVB in a relatively young patient without heart disease, an electrophysiology study should be considered to rule out concealed extrasystoles when typical diagnostic ECG findings are missing because these extrasystoles may be amenable to pharmacological therapy omitting unnecessary implantation of a pacemaker (43).

## Conclusion

The correct diagnosis of second-degree AVB is basically an exercise in logic based on the clinical application of relatively simple definitions and knowledge of the various diagnostic pitfalls. Mobitz type II AVB is the most commonly misdiagnosed arrhythmia mostly because of the widespread use of disparate definitions (45–48). Many of the problems are best understood by reviewing the historical aspects of the second-degree AV block (4). To avoid the commonest mistake, one must always remember that the diagnosis of type II block requires a stable sinus rate. Ignoring the importance of sinus slowing may lead to an erroneous diagnosis of type II block in common clinical situations, such as AVB, during normal sleep or sleep apnea and in athletes (35, 36).

## References

[1] Upshaw CB Silverman ME. John Hay: discoverer of type II atrioventricular block. Clin Cardiol. (2000) 23:869–71. 10.1002/clc.4960231118 11097138 PMC6655013

[2] Mobitz W. Uber die unvollstandige stozzrung der erregungsuberleitung zwischen vorhof und kammer des menschlichen herzems. Z Ges Exp Med. (1924) 41:180–237. 10.1007/BF02758773

[3] Upshaw CB Jr Silverman ME. Woldemar Mobitz: early twentieth century expert on atrioventricular block. Clin Cardiol. (2009) 32:E75–7. 10.1002/clc.20377 19816971 PMC6653676

[4] Barold SS. Problems in the definition of Mobitz type II atrioventricular block. Historical perspective. J Electrocardiol. (2023) 79:122–3. 10.1016/j.jelectrocard.2023.03.086 37084494

[5] El-Sherif N Scherlag BJ LAzzara R. Pathophysiology of second degree atrioventricular block: a unified hypothesis. Am J Cardiol. (1975) 35:421–34. 10.1016/0002-9149(75)90036-3 1121954

[6] Barold SS Hayes DL. Second-degree atrioventricular block: a reappraisal. Mayo Clin Proc. (2001) 76:44–57. 10.4065/76.1.44 11155413

[7] Barold SS. The Chicago School of electrocardiography and second-degree atrioventricular block: an historical perspective. Pacing Clin Electrophysiol. (2001) 24:138–46. 10.1046/j.1460-9592.2001.00138.x 11270692

[8] Clark BA Prystowsky EN. Electrocardiography of atrioventricular block. Cardiol Clin. (2023) 41:307–13. 10.1016/j.ccl.2023.03.007 37321683

[9] Clark BA Prystowsky EN. Electrocardiography of atrioventricular block. Card Electrophysiol Clin. (2021) 13:599–605. 10.1016/j.ccep.2021.07.001 34689889

[10] WHO/ISC Task Force. Definition of terms related to cardiac rhythm. Am Heart J. (1978) 95:796–80. 10.1016/0002-8703(78)90512-4 655094

[11] Surawicz B Uhley H Borun R Laks M Crevasse L Rosen K The quest for optimal electrocardiography. Task force I: standardization of terminology and interpretation. Am J Cardiol. (1978) 41:130–45. 10.1016/0002-9149(78)90147-9 622995

[12] Barold SS Friedberg HD. Second-degree atrioventricular block: a matter of definition. Am J Cardiol. (1974) 33:311–5. 10.1016/0002-9149(74)90297-5 4810033

[13] Massie B Scheinman MM Peters R Desai J Hirschfeld D O’Young J. Clinical and electrophysiologic findings in patients with paroxysmal slowing of the sinus rate and apparent Mobitz type II atrioventricular block. Circulation. (1978) 58:305–14. 10.1161/01.CIR.58.2.305 668079

[14] Alboni P Holz A Brignole M. Vagally mediated atrioventricular block: pathophysiology and diagnosis. Heart. (2013) 99:904–8. 10.1136/heartjnl-2012-303220 23286970

[15] Barold SS. 2:1 Atrioventricular block: order from chaos. Am J Emerg Med. (2001) 19:214–7. 10.1053/ajem.2001.21715 11326349

[16] Elkin A Goldschlager N. Atrioventricular block with 2:1 conduction: where is the block, and how should it be managed? JAMA Intern Med. (2013) 173:335–7. 10.1001/jamainternmed.2013.3182a 23400335

[17] Naono H Takeda R Masuyama H Kawano J Naono-Nagatomo K Ishida Y. Case of reversible Mobitz type II atrioventricular block after the use of injectable antipsychotics. Clin Case Rep. (2022) 10:e05326. 10.1002/ccr3.5326 35127093 PMC8795839

[18] Kusumoto FM Schoenfeld MH Barrett C Edgerton JR Ellenbogen KA Gold MR 2018 ACC/AHA/HRS guideline on the evaluation and management of patients with bradycardia and cardiac conduction delay: a report of the American College of Cardiology/American Heart Association Task Force on Clinical Practice Guidelines and the Heart Rhythm Society. Circulation. (2019) 140:e382–482. 10.1161/CIR.0000000000000628 30586772

[19] Glikson M Nielsen JC Kronborg MB Michowitz Y Auricchio A Barbash IM 2021 ESC guidelines on cardiac pacing and cardiac resynchronization therapy. Eur Heart J. (2021) 42:3427–520. 10.1093/eurheartj/ehab364 34586378

[20] Freeman G Hwang MH Danoviz J Moran JF Gunnar RM. Exercise induced Mobitz type II second degree AV block in a patient with chronic bifascicular block (right bundle branch block and left anterior hemiblock). J Electrocardiol. (1984) 17:409–12. 10.1016/S0022-0736(84)80079-5 6209355

[21] Bonikowske AR Barout A Fortin-Gamero S Lara MIB Kapa S Allison TG. Frequency and characteristics of exercise-induced second-degree atrioventricular block in patients undergoing stress testing. J Electrocardiol. (2019) 54:54–60. 10.1016/j.jelectrocard.2019.03.009 30925274

[22] Lescure M Dechandol AM Lagorce P Marot M Goutner C Donzeau JP. Blocs auriculo-ventriculaires d'effort. A propos de 62 cas [effort-induced atrioventricular block. Apropos of 62 cases]. Ann Cardiol Angeiol (Paris). (1995) 44:486–92. (French). 8745658

[23] Woelfel AK Simpson RJ Jr Gettes LS Foster JR. Exercise-induced distal atrioventricular block. J Am Coll Cardiol. (1983) 2:578–81. 10.1016/S0735-1097(83)80288-5 6875122

[24] Lange HW Ameisen O Mack R Moses JW Kligfield P. Prevalence and clinical correlates of non-Wenckebach, narrow-complex second-degree atrioventricular block detected by ambulatory ECG. Am Heart J. (1988) 115:114–20. 10.1016/0002-8703(88)90526-1 3336966

[25] Barold SS Jais P Shah DC Takahashi A Haissaguerre M Clementy J. Exercise-induced second-degree AV block: is it type I or type II? J Cardiovasc Electrophysiol. (1997) 8:1084–6. 10.1111/j.1540-8167.1997.tb00633.x 9300307

[26] Bansal R Rathi C Lokhandwala Y. Where is the level of atrioventricular block? Circulation. (2020) 142:1684–6. 10.1161/CIRCULATIONAHA.120.050344 33104401

[27] Armstrong EJ Dubey A Scheinman MM Badhwar N. Lithium-associated Mobitz II block: case series and review of the literature. Pacing Clin Electrophysiol. (2011) 34:e47–51. 10.1111/j.1540-8159.2010.02773.x 20456646

[28] Tay YL Tay KH Ying J Tor PC. Lithium-induced symptomatic second-degree heart block: a case report. Asian J Psychiatr. (2021) 55:102486. 10.1016/j.ajp.2020.102486 33264720

[29] Serinken M Karcioglu O Korkmaz A. Rarely seen cardiotoxicity of lithium overdose: complete heart block. Int J Cardiol. (2009) 132:276–8. 10.1016/j.ijcard.2007.08.058 18068832

[30] Jaffe CM. First-degree atrioventricular block during lithium carbonate treatment. Am J Psychiatry. (1977) 134:88–9. 10.1176/ajp.134.1.88 831549

[31] Martin CA Piascik MT. First degree A-V block in patients on lithium carbonate. Can J Psychiatry. (1985) 30:114–6. 10.1177/070674378503000206 3922608

[32] Zeltser D Justo D Halkin A Rosso R Ish-Shalom M Hochenberg M Drug-induced atrioventricular block: prognosis after discontinuation of the culprit drug. J Am Coll Cardiol. (2004) 44:105–8. 10.1016/j.jacc.2004.03.057 15234417

[33] Kariyanna PT Jayarangaiah A Al-Sadawi M Ahmed R Green J Dubson I A rare case of second degree Mobitz type II AV block associated with cocaine use. Am J Med Case Rep. (2018) 6(7):146–8. 10.12691/ajmcr-6-7-7

[34] Roca-Luque I Francisco-Pasqual J Oristrell G Rodríguez-García J Santos-Ortega A Martin-Sanchez G Flecainide versus procainamide in electrophysiological study in patients with syncope and wide QRS duration. JACC Clin Electrophysiol. (2019) 5:212–9. 10.1016/j.jacep.2018.09.015 30784693

[35] Barold SS Padeletti L. Mobitz type II second-degree atrioventricular block in athletes: true or false? Br J Sports Med. (2011) 45:687–90. 10.1136/bjsm.2008.047365 18801768

[36] Barold SS. Mobitz type II second-degree atrioventricular block during sleep: true or false? Herzschrittmacherther Elektrophysiol. (2023) 34:226–8. 10.1007/s00399-023-00959-y 37540286

[37] El-Sherif N Aranda J Befeler B Lazzara R. Atypical Wenckebach periodicity simulating Mobitz type II AV block. Brit Heart J. (1978) 40:1376–83. 10.1136/hrt.40.12.1376 737095 PMC483582

[38] Steinbrecher UP Fitchett DH. Torsade de pointes. A cause of syncope with atrioventricular block. Arch Intern Med. (1980) 140:1223–6. 10.1001/archinte.1980.00330200099027 7406620

[39] Stankovic I Putnikovic B Neskovic AN. Torsades de pointes complicating complete heart block with QT interval prolongation. Acta Cardiol. (2016) 71(5):627. 10.1080/AC.71.5.5.3167513 27695027

[40] Moroe K Saku K Tashiro N Hiroki T Arakawa K. “Torsades de pointes” and atrioventricular block. Clin Cardiol. (1988) 11(1):9–13. 10.1002/clc.4960110111 3349663

[41] Antonelli D Rozner E Freedberg NA Turgeman Y. Torsade de pointes—a cause of Morgagni–Adam–Stokes attacks in patients with complete atrioventricular block. Harefuah. (2008) 147:204–6. (Hebrew). 18488859

[42] Langendorf R Mehkmann JS. Blocked (non-conducted) A-V nodal premature systoles imitating first and second degree A-V block. Am Heart J. (1947) 34:500–6. 10.1016/0002-8703(47)90528-0 20266465

[43] Rosen KM Rahimtoola SH Gunnar RM. Pseudo A-V block secondary to premature non-propagated His bundle depolarizations: documentation by His bundle electrocardiography. Circulation. (1970) 42:367–73. 10.1161/01.CIR.42.3.367 5451223

[44] Ho R Ruskin J Ng CY. Concealed His extrasystoles: a masquerader of atrioventricular block. Heart Rhythm Case Rep. (2022) 8:727–9. 10.1016/j.hrcr.2022.07.004 PMC9811016 36618590

[45] Phibbs BP. Diagnosis of complex forms of AV block: some tricks, some booby traps. In: Phibbs BP, editor. Advanced ECG. Boards and Beyond. Boston, MA: Little, Brown (1997). p. 110–24.

[46] Gillespie ND Brett CT Morrison WG Pringle SD. Interpretation of the emergency electrocardiogram in junior hospital doctors. J Accid Emerg Med. (1996) 13:395–7. 10.1136/emj.13.6.395 8947796 PMC1342806

[47] Lever NA Larsen PD Dawes M Wong A Harding SA. Are our medical graduates in New Zealand safe and accurate in electrocardiography interpretation? N Z Med J. (2009) 122:9–15. 19448769

[48] Elsava D Dhillon SS Berger J Homer P. Bergmann S interpretation of electrocardiograms by first-year residents: the need for change. J Electrocardiol. (2009) 42:693–7. 10.1016/j.jelectrocard.2009.07.020 19740482

